# The Status of Stress Myocardial Perfusion Imaging Using 99mTc Pharmaceuticals in Japan: Results from a Nationwide Survey

**DOI:** 10.22038/aojnmb.2018.10477

**Published:** 2018

**Authors:** Ryuto Otsuka, Yosuke Miyazaki, Narumi Kubo, Mio Kawahara, Jun Takaesu, Kazuki Fukuchi

**Affiliations:** Department of Medical Physics and Engineering, Course of Health Science, Osaka University Graduate School of Medicine, Osaka, Japan

**Keywords:** Myocardial perfusion imaging, Nuclear medicine, SPECT

## Abstract

**Objective(s):**

To appropriately use one-day myocardial perfusion imaging (MPI) with^ 99m^Tc radiopharmaceuticals (i.e. to avoid shine-through artifacts), injection doses need to be optimized and dose ratios between the 1^st^ and 2^nd^ scans should be maintained at ≥ 3. However, the current state of practice in Japan is unclear. Thus, the aim of this study was to clarify the details of MPI protocols using ^99m^Tc radiopharmaceuticals in Japan.

**Methods:**

A nationwide survey was conducted in June and July 2016. Questionnaires about stress MPI protocols using ^99^^m^Tc radiopharmaceuticals were sent to 641 nuclear medicine facilities.

**Results:**

Responses were received from 246 facilities. One-day protocols were used in 97.1% of the facilities. The most common injection dose ratio was 2.5. Only 18.2% of facilities achieved the recommended injection dose ratio. Stress-only protocols were performed in only 1.7% of facilities; the primary reasons for not performing stress-only protocols were as follows: 1) “The reading-physician cannot interpret the image just after the first scan,” and 2) “Preparation of radiopharmaceuticals and scan arrangements turn out to be complicated.”

**Conclusion:**

Approximately 80% of nuclear medicine facilities do not follow the recommended injection dose ratio. Stress-only protocols are ideal, but are performed at very few facilities. Both optimization and standardization of stress MPI protocols using ^99m^Tc radiopharmaceuticals are needed in Japan.

## Introduction

Myocardial perfusion imaging (MPI) is one of the most popular diagnostic methods for coronary artery disease (CAD) (1); it can also be used to stratify risk and evaluate treatment efficacy in patients with suspected or known CAD (2-5). Fifteen to twenty million procedures are performed annually worldwide; however, the ionizing radiation generated during MPI is a cause for concern (6). Therefore, eight best practices (EBP) were proposed by the International Atomic Energy Agency (IAEA) to optimize MPI radiation doses (7). In this statement, several proposals regarding radiopharmaceutical use for one-day stress protocols were mentioned. For example, to avoid shine-through artifacts (i.e. where residual radioactivity from the first injection interferes with the interpretation of images from the second injection), the IAEA statement recommends that the second injection dose is three or more times that of the first injection (i.e. injection dose ratio ≥ 3) (7). In addition, to reduce radiation doses by up to 80%, it is recommended that stress-only protocols be the directive by which physicians perform cardiac stress tests, without complementary resting scans (7). However, the state of practice of MPI radiopharmaceutical use in Japan is unclear.

Thus, the aim of our study was to clarify the current stress MPI protocols using ^99m^Tc radiopharmaceuticals in Japan.

## Methods

This nationwide survey was carried out alongside a previously reported study (8). Briefly, we identified 1,249 facilities conducting nuclear medicine examinations in Japan via a Japanese trade journal, published in March 2016. We then checked the websites of these facilities and found 641 clearly advertising MPI on their homepage. Questionnaires regarding stress MPI were sent to these facilities in June and July 2016; the survey was closed at the end of September 2016.

All questions assumed that patients receiving stress MPI using ^99m^Tc radiopharmaceuticals were adults with a sinus rhythm and standard Japanese body weight (60 kg). The questionnaire included the following five questions:

1. Which types of stress protocols does your facility perform: one-day, two-day, or both?

2. Which types of one-day protocols does your facility perform: stress-first, rest-first, or both?

3. What doses of ^99m^Tc radiopharmaceuticals does your facility use for the one-day protocol? Provide the injection doses for the first and second scans, separately.

4. How many interval-hours does your facility apply between the first and second scans?

5. In your facility, is it possible to perform the stress-only protocol? Choose answers from the following five options (multiple answers are acceptable).

6. Yes, we already perform it.

7. It is possible, but we do not perform it yet.

8. It is difficult to perform it because radiopharmaceutical preparations and scan arrangements are complicated.

9. It is difficult to perform it because the reading-physician cannot interpret the image just after the first scan.

10. Others (free responses are accepted).

## Results

We obtained responses from 431 facilities out of 641 (response rate: 67%). Of those 431 facilities, 185 did not use ^99m^Tc radiopharmaceuticals but rather ^201^TlCl for stress MPI. Thus, valid responses on stress MPI protocols using ^99m^Tc radiopharmaceuticals were obtained from 246 facilities. 

**Figure 1:**
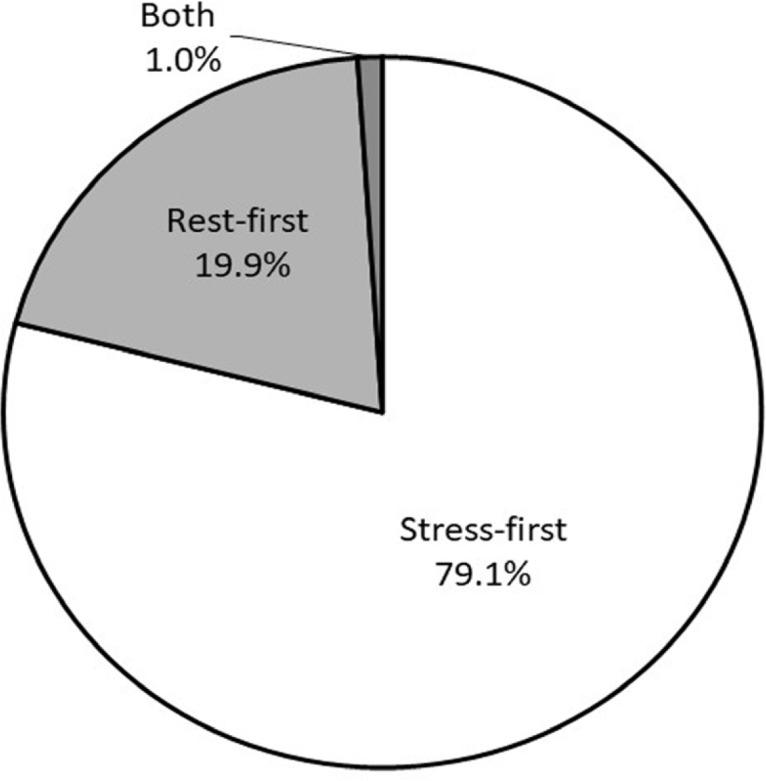
The percentage of facilities that performed stress- and rest- first stress myocardial perfusion imaging protocols

Of the 244 facilities that answered question 1, 237 (97.1%) performed the one-day protocol, 5 (2.1%) performed the two-day protocol, and 2 (0.8%) performed both protocols. The types of one-day protocols performed by the facilities are shown in Figure 1. Of the 191 facilities that answered question 2, 151 performed the stress-first protocol, 38 performed the rest-first protocol, and 2 performed both protocols.

The injection doses of ^99m^Tc radiopharmaceuticals used by the facilities are shown in Table 1. Of the 226 facilities that answered question 3 (20 failed to answer this question), 103 (45.6%) used doses of 296 MBq and 740 MBq for the first and second scans, respectively (this combination was most popular in both the stress- and rest- first protocols), and 29 (12.8%) used doses of 370 MBq and 740 MBq for the first and second scans, respectively. The maximum injection dose-combination was 444 MBq and 1,110 MBq for the first and second scans, respectively (total 1,554 MBq), and the minimum injection dose-combination was 120 MBq and 360 MBq for the first and second scans, respectively (total 480 MBq); representing a maximum-minimum dose difference of 3.2 times. 

**Figure 2:**
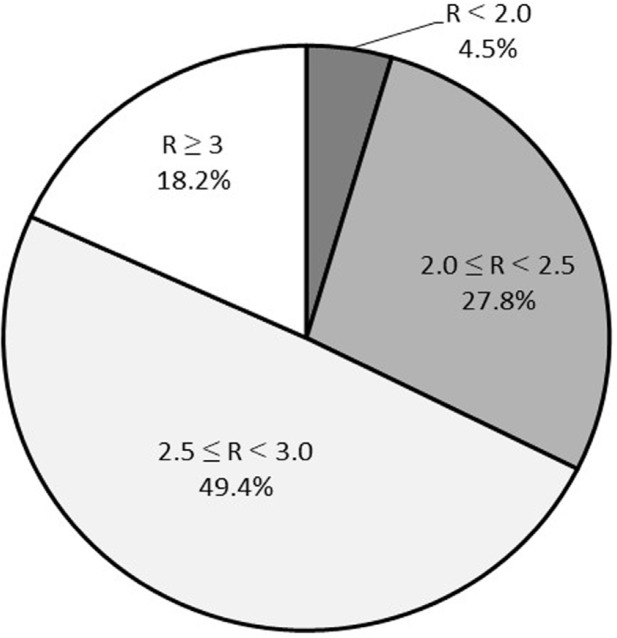
The percentage of facilities that used different injection dose ratios

Figure 2 shows the percentage of facilities using different injection dose ratios. The most common injection dose ratio ranged from ≥2.5 to <3.0, which was lower than the recommended by the guidelines. Only 18.2% of facilities followed the recommended injection dose ratio.

**Figure 3.**
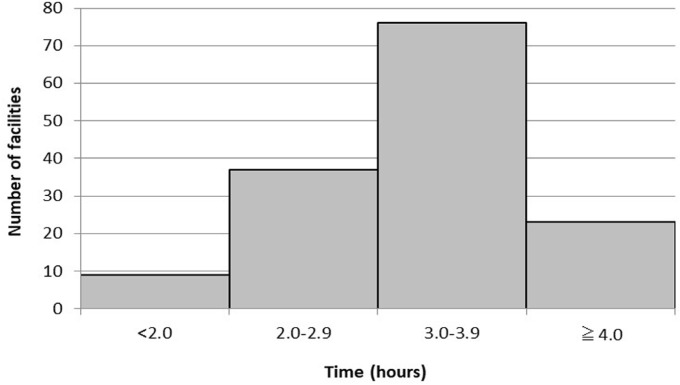
The distribution of the time intervals between the first and second scans in the stress-first protocol

**Figure 4:**
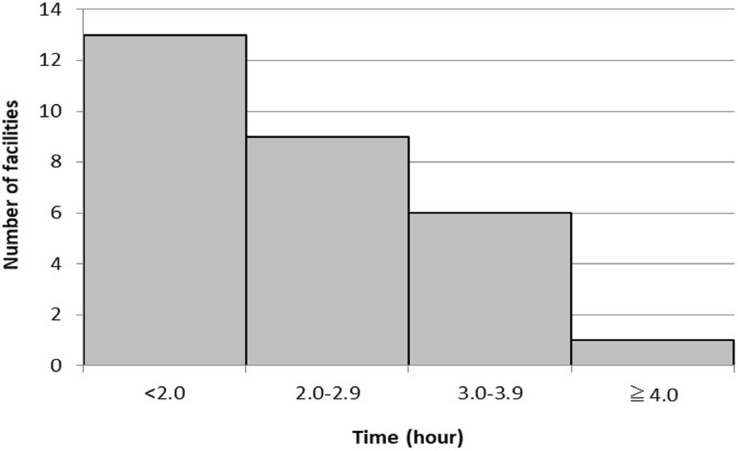
The distribution of the time intervals between the first and second scans in the rest-first protocol

The time intervals applied by the facilities between the first and second scans are shown in Figure 3 and 4. Regarding question 4, 145 facilities answered for stress-first protocols, and 29 facilities answered for rest-first protocols. For stress-first protocols, answers (in order of popularity) included 3.0–3.9 hours (76/145, 52.4%), 2.0–2.9 hours (37/145, 25.5%), and ≥4.0 hours (23/145, 15.9%). For rest-first protocols, answers (in order of popularity) included <2.0 hours (13/29, 44.8%), 2.0–2.9 hours (9/29, 31.0%), and 3.0–3.9 hours (6/29, 20.7%). 

**Figure 5.**
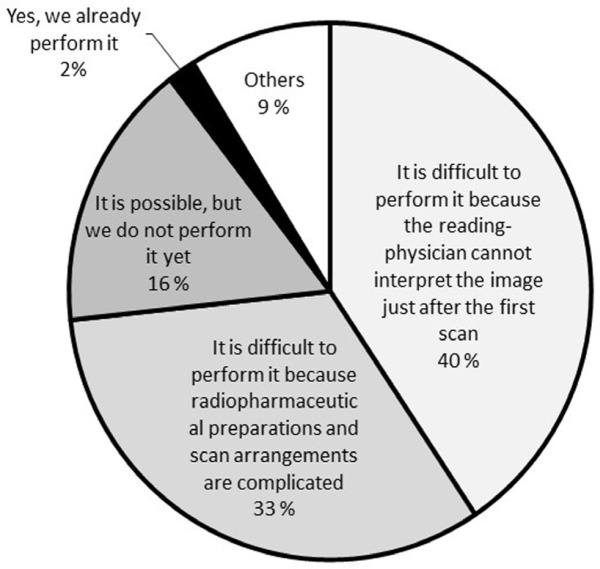
The percentage of answers indicating the possibility of performing stress-only protocols, and the reasons for these responses

Finally, with regards to the possibilities for facilities to perform stress-only protocols (question 5), the principal answers are shown in Figure 5. As each facility chose multiple reasons, percentages represent the total number of answers (352) as opposed to the total number of facilities. Answers (in order of popularity) included, “It is difficult to perform it because the reading-physician cannot interpret the image just after the first scan” (143/352, 40.6%), “It is difficult to perform it because preparation of radiopharmaceuticals and scan arrangements are complicated” (115/352, 32.7%), “It is possible, but we do not perform it yet” (57/352, 16.2%), “Yes, we already perform it” (6/352, 1.7%), and “Others” (31/352, 8.8%). The specific reasons for choosing “Others” (in order of popularity) included, “It is impossible due to performing the rest-first protocol” (12/31) and “The feasibility of the stress-only protocol has not been established yet” (3/31).

## Discussion

In 2013, the International Atomic Energy Agency nuclear cardiology protocols cross-sectional study (INCAPS) evaluated global MPI protocols (9), and data from only 9 patients were provided from a single facility in Japan. Thus, INCAPS is not reflective of the actual status of MPI in Japan (10). To the best of our knowledge, this is the first survey providing an overview of the current state of stress MPI practice using ^99m^Tc radiopharmaceuticals in Japan.

Our survey revealed that stress-first protocols are major one-day protocols in Japan. According to the INCAPS results, similar trends can be seen in Africa (89.9%), Asia (68.2%), and Europe (84.4%); however, in other regions, the rest-first protocol is more popular (11). For example, the percentage-use of stress-first protocols in Latin America, North America, and Oceania were 40.7%, 7.7%, and 21.0%, respectively (11); thus, there are no substantial differences between the two protocols with regards to the nature of perfusion abnormalities. There are some advantages and disadvantages in choosing the type of one-day protocol. For example, the rest-first protocol is better than the stress-first sequence for detecting the reversibility of stress-induced perfusion deficits (12, 13); thus, more than 90% of North American facilities perform the rest study first (14). However, there is no need for a rest study when the rest scan is normal, and staff experience considerably higher radiation exposure with previous rest studies. Moreover, an exercise/rest sequence seems to be more acceptable when stress ^201^TlCl is used on same day; according to our previous study, 38.6% of facilities that perform stress MPI with ^99m^Tc radiopharmaceuticals also use ^201^TlCl for stress testing (8). In such facilities, a stress-first protocol may be more suitable, as the stress testing setup is similar for both tracers. This may explain the predominance of stress-first studies in Japan. 

**Table 1:** The popular injection doses of stress myocardial perfusion imaging using ^99m^Tc radiopharmaceuticals in Japan

**Order**	**First injected dose (MBq)**	**Second injected dose (MBq)**	**Total injected dose (MBq)**	**Number of facilities**
1	296	740	1,036	103
2	370	740	1,110	29
3	185	555	740	13
3	296	592	888	13
5	200	800	1,000	5
6	247	493	740	3
6	300	740	1040	3

R = injection dose ratio

The most popular injection dose combination was 296 MBq and 740 MBq for the first and second scans, respectively. The injection dose ratio for this combination is 2.5, which is not in accordance with the recommended dose ratio of ≥ 3; this recommended ratio was achieved by only 18 of 140 facilities (12.9%) in the stress-first protocol, and 14 of 36 facilities (38.9%) in the rest-first protocol. Therefore, only 32 of 176 facilities (18.2%) achieved the recommended dose ratio in total, indicating that approximately 80% of MPI examinations in Japan were performed inappropriately in terms of cancelling shine-through artifacts. By contrast, the recommended injection dose ratio was achieved in an average of 58%, 38%, 65%, 39%, 15%, 44%, and 44% of facilitates in Africa, Asia, Europe, Latin America, North America, Oceania, and worldwide, respectively (7). The achievement rates in Japan and North America were considerably lower than those in other regions. In rest-first protocols, shine-through artifacts cause underestimation of ischemia in stress imaging. On the other hand, in stress-first protocols, shine-through artifacts can affect rest imaging and underestimate the fill-in phenomena. Guidelines recommend that the activity of the second injection should be at least three times higher than that of the first injection, and that the optimal time interval between the two images should be more than two hours to avoid shine-through artifacts (15, 16). However, this protocol was determined in the late 1980s using a limited number of patient data (17) and no subsequent detailed studies have been carried out to check the feasibility of this magnification. In North America (rest-first dominant) and Japan (stress-first dominant), less than 20% of facilities conform to this magnification guideline, but no significant operational issues have been highlighted. Thus, an appropriate injection dose ratio to avoid shine though artifacts has not yet been identified, and further examination is needed for clarification. 

Our survey revealed a difference of approximately 3 times between the maximal (total 1,554 MBq) and minimal (total 480 MBq) injection doses in the same stress MPI using ^99m^Tc radiopharmaceuticals. Ionizing radiation exposure reaches 3 times when the injection dose is tripled, and this disparity cannot be overlooked. The facility managing the minimal injection dose uses a cadmium zinc telluride camera for cardiac imaging, which is an effective dose-reduction strategy (18). However, there are currently few cadmium zinc telluride cameras in Japan; the introduction of other camera-based technologies should be promoted to reduce injection doses in facilities that use conventional Angar cameras (19).

The percentage of facilities performing the stress-only protocol in Japan was only 2.4% (6 of 246 facilities). The mean percentage of facilities performing stress-only protocols worldwide is 11.9% (11). It seems that Japan has lower penetration rates of stress-only protocols compared to other countries. Two reasons for this were particularly obvious through our survey; firstly, reading-physicians cannot interpret the image just after the first scan and, secondly, radiopharmaceutical preparations turn out to be complicated. In likelihood, other countries face similar problems in adopting stress-only protocols in routine work. Thus, it is necessary to change the workflow of nuclear medicine examinations to overcome these problems. If stress images are completely normal, subsequent rest imaging can be avoided to dramatically reduce radiation doses (7). Therefore, we should consider increasing the introduction of stress-only protocols. Fortunately, stress-first protocols are popular in Japan. Thus, resistance to performing stress-first protocols is lower than that to performing rest-first protocols. 

***Study Limitations***

Firstly, our questionnaire assumed that the surveyed facilities tested patients with a standard Japanese body weight; however, this data was not extracted from actual examinations. Therefore, our survey is different from the INCAPS survey that collected actual data over a given period.

Secondly, many facilities in Japan use ready-made syringes containing ^99m^Tc-labeled radiopharmaceuticals at a fixed radiation dose. However, in such cases, the actual injected dose (at the time point of injection) is uncertain. In this study, we could not distinguish the data of cases that used ready-made syringes from those of cases that used in-house preparations. 

## Conclusion

In Japan, standard stress MPI protocols using ^99m^Tc radiopharmaceuticals are one-day, stress-first methods, with 2.5 times injection dose ratios and 2- to 3- hour scan intervals. Approximately 80% of facilities do not reach the recommended injection dose ratio and there are relatively large variations in the total injected doses. We need to adhere to a national standard protocol for stress MPI using ^99m^Tc radiopharmaceuticals. 
